# Using customs data to understand overlooked trade in non‐CITES birds between Africa and Asia

**DOI:** 10.1111/cobi.70265

**Published:** 2026-03-23

**Authors:** Alisa Davies, Astrid A. Andersson, Rowan O. Martin, Sam Inglis, Caroline Dingle

**Affiliations:** ^1^ World Parrot Trust Hayle UK; ^2^ School of Biological Sciences The University of Hong Kong Hong Kong Hong Kong; ^3^ ADM Capital Foundation Hong Kong Hong Kong; ^4^ FitzPatrick Institute of African Ornithology University of Cape Tow Cape Town South Africa; ^5^ Biology Department Capilano University North Vancouver British Columbia Canada

**Keywords:** CITES, *Crithagra*, international trade, songbird, trade patterns, wildlife trade, Aves canoras, CITES, comercio internacional, comercio de vida silvestre, *Crithagra*, patrones comerciales, 国际贸易, 鸣禽, 贸易模式, 《濒危野生动植物种国际贸易公约》(CITES), 野生动物贸易, 丝雀属(*Crithagra*)

## Abstract

The international trade in live birds poses risks to animals, people, and biodiversity. To effectively mitigate these risks, decision‐makers require information on the volume, dynamics, and direction of trade. Despite Africa once being the largest exporter of birds by region, very little data exist on recent trade in live birds not listed on the appendices of the Convention on International Trade in Endangered Species of Wild Fauna and Flora (CITES). We used UN Comtrade data to explore trade in non‐CITES birds from African countries to key Asian hubs for wildlife trade, Hong Kong and Singapore, from 2006 to 2020. We supplemented these data with species‐specific data obtained from the Hong Kong government for 2015–2020 to further understand the taxonomic composition of recent imports. Over a million non‐CITES birds were imported to Hong Kong and Singapore from 2006 to 2020. Africa accounted for an increasing proportion of these imports, with West African countries, particularly Mali, playing an increasingly important role in recent years. Import data from the Hong Kong government indicated that canaries (*Crithagra* spp.) dominated these imports, including species that have been heavily traded for decades and may be experiencing declines in the wild. Although Comtrade data can provide insights into international wildlife trade, particularly for species that are otherwise difficult to monitor, we propose that its usefulness could be improved by increasing the taxonomic specificity of harmonized system codes to include lower taxonomic levels. To mitigate biosecurity risks and negative impacts on wild populations associated with the large‐scale trade in wild birds that we found, we propose that importing countries broaden restrictions on imports of live birds. Specifically, we recommend that importing countries restrict imports from countries unable to demonstrate the legal acquisition of birds or that do not have robust quarantine and surveillance systems for pathogens of human and animal health concern.

## Abstract (es)

El uso de datos aduanales para comprender el comercio no registrado de aves no incluidas en la CITES entre África y Asia

**Resumen:**

El comercio internacional de aves vivas plantea riesgos para los animales, las personas y la biodiversidad. Para mitigar eficazmente estos riesgos, los responsables de la toma de decisiones necesitan información sobre el volumen, la dinámica y la dirección del comercio. A pesar de que África fue en su día el mayor exportador de aves por región, existen muy pocos datos sobre el comercio reciente de aves vivas que no figuran en los apéndices de la Convención sobre el Comercio Internacional de Especies Amenazadas de Fauna y Flora Silvestres (CITES). Utilizamos datos de UN Comtrade para analizar el comercio de aves no incluidas en la CITES desde países africanos hacia los principales centros asiáticos de comercio de vida silvestre, Hong Kong y Singapur, entre 2006 y 2020. Complementamos estos datos con información específica por especie obtenida del gobierno de Hong Kong para el período 2015‐2020, con el fin de comprender mejor la composición taxonómica de las importaciones recientes. Entre 2006 y 2020 se importaron más de un millón de aves no incluidas en la CITES a Hong Kong y Singapur. África representó una proporción cada vez mayor de estas importaciones, y los países de África Occidental, en particular Malí, han desempeñado un papel cada vez más importante en los últimos años. Los datos de importación del gobierno de Hong Kong indicaron que los canarios (*Crithagra* spp.) dominaban estas importaciones, incluidas especies que han sido objeto de un intenso comercio durante décadas y que podrían estar experimentando disminuciones en su estado silvestre. Si bien los datos de Comtrade pueden ofrecer información sobre el comercio internacional de fauna silvestre, especialmente en el caso de especies que, de otro modo, serían difíciles de monitorear, proponemos que se mejore su utilidad aumentando la especificidad taxonómica de los códigos del Sistema Armonizado para incluir niveles taxonómicos inferiores. Para mitigar los riesgos de bioseguridad y los impactos negativos en las poblaciones silvestres asociados con el comercio a gran escala de aves silvestres que hemos detectado, proponemos que los países importadores amplíen las restricciones a la importación de aves vivas. Específicamente, recomendamos que los países importadores restrinjan las importaciones procedentes de países que no puedan demostrar la adquisición legal de las aves o que no cuenten con un sistema sólido de cuarentena y vigilancia de patógenos que representen un riesgo para la salud humana y animal.

## 利用海关数据揭示非洲与亚洲间未列入《濒危野生动植物种国际贸易公约》鸟类被忽视的贸易

**【摘要】** 活体鸟类国际贸易对动物、人类及生物多样性构成风险。决策者需掌握贸易规模、动态及流向信息, 才能有效缓解相关风险。尽管非洲曾是全球最大的鸟类出口地区, 但关于未列入《濒危野生动植物种国际贸易公约》(CITES)附录的活体鸟类近期贸易的数据仍十分匮乏。我们利用联合国商品贸易统计数据库(UN Comtrade), 追踪了2006至2020年间从非洲国家向亚洲野生动物贸易枢纽(香港和新加坡)出口非CITES鸟类的贸易情况。我们补充了香港政府提供的2015‐2020年物种特异的数据, 以深入了解近期进口鸟类的类群组成。2006至2020年间, 香港和新加坡共进口超过一百万只非CITES鸟类。非洲在进口来源中的占比持续上升, 其中西非国家(尤其是马里)近年扮演着日益重要的角色。香港政府的进口数据显示丝雀属物种(*Crithagra* spp.)占据主导地位, 其中包括数十年来被大量贸易且野外种群可能正在减少的物种。尽管联合国商品贸易统计数据库能为国际野生动植物贸易提供重要信息, 尤其是针对难以用其他手段监测的物种, 但我们建议仍可通过提高协调制度商品编码的类群特异性(纳入更低分类层级)来增强其实用性。为减缓大规模野生鸟类贸易带来的生物安全风险及对野生种群的负面影响, 我们建议进口国扩大对活体鸟类进口的限制范围。具体而言, 我们建议应对无法证明鸟类合法来源的国家, 或对人类及动物健康病原体缺乏严格检疫和监测的国家实施进口管制。**【翻译:胡怡思;审校:聂永刚】**

## INTRODUCTION

The legal international trade of live animals for exotic pets involves millions of animals annually (Bush et al., 2014; Harfoot et al., 2018; Lockwood et al., 2019). Birds are one of the most frequently traded and diverse taxonomic groups (Bush et al., 2014; Hughes et al., 2021; Ribeiro et al., 2019; Scheffers et al., 2019). At least a third of the world's bird species (33.9%) are traded (Butchart, 2008; Nijman, 2010; Scheffers et al., 2019), and a greater proportion of traded birds are sourced from the wild compared with other taxa such as mammals or reptiles (Bush et al., 2014).

Overexploitation for trade is a major threat to bird conservation (Birdlife International, 2023), causing the decline of wild populations of heavily traded species in Africa (Khelifa et al., 2017; Martin, 2018a), Southeast Asia (Harris et al., 2017), and South America (Alves et al., 2013). Official trade figures are often snapshots of trade and do not consider the turnover of animals sold and mortality throughout the supply chain, which also leads to underestimates of the volume of birds taken from the wild (Lambert et al., 2025; Leader‐Williams & Tibanyenda, 1996; Wyatt et al., 2022). Biodiversity in importing and transit countries is threatened by live species introduced via trade (Cardador et al., 2019; Lockwood et al., 2019), and human, livestock, and wildlife health is at risk from zoonotic diseases, including highly pathogenic avian influenza (H5N1), Newcastle disease, and psittacine beak and feather disease (Fogell et al., 2018; Karesh et al., 2007). Achieving sustainable and safe trade is challenged by inadequate monitoring of trade and its impacts on wild populations, a lack of clear policies and processes, and resources for regulation (Gilardi, 2006; Hughes et al., 2021; Shepherd et al., 2023). Appropriate policy responses and enforcement interventions to mitigate risks depend on a robust understanding of the volume, composition, and dynamics of recent trade.

The Convention on International Trade in Endangered Species of Wild Fauna and Flora (CITES) is the principal multilateral framework through which international trade in wildlife is regulated. Parties to the Convention are required to monitor trade in species listed under the appendices of CITES, and these data are publicly accessible. However, many traded species are not listed in the appendices (hereafter, *non‐CITES species*) (e.g., ∼1.4% of songbird species, order Passeriformes, are listed in the CITES appendices), and, as a result, detailed, readily accessible, and current data on a large proportion of wildlife trade are lacking (Juergens et al., 2021). Trade in non‐CITES birds has been investigated and evaluated using market and social media surveys (e.g., Chiok & Chng, 2021; Davies, Nuno, et al., 2022; Harris et al., 2015). However, these methods typically provide a snapshot of trade within specific jurisdictions for particular modes of sale and often focus on availability within consumer markets. As a result, the volume of international trade in non‐CITES species can be underestimated in analyses and discussions of international wildlife trade (Davies, Nuno, et al., 2022; Janssen & Shepherd, 2018), impeding effective management and the development of evidence‐based policy.

Recently, researchers have turned to the United Nations International Trade Statistics Database (UN Comtrade), which aggregates data on commodities traded between UN member parties, including live animals for the pet trade (Andersson et al., 2021; Gephart & Pace, 2015), for data on the wider scope of species in trade. The system captures trade in all species, including those not listed in the appendices of CITES, providing an opportunity to gather data on volumes and directions of trade for non‐CITES species. The database and collection protocol are designed for taxation purposes and therefore have several limitations for understanding important facets of wildlife trade (see “Opportunities and challenges of UN Comtrade data” and Chan et al. [2015] and Andersson et al. [2021]). These include most data being reported at higher taxonomic levels (i.e., order or higher) in most cases and no indication of source (e.g., captive bred or wild). However, combined with ancillary information, including internal government data and knowledge of avicultural and bird trapping practices in exporting countries, useful inferences of trade patterns can be made.

Historically, African countries have played a major role in the international bird trade, with Guinea, Mali, and Senegal collectively accounting for 70% of all bird exports reported to CITES from 1995 to 2005 (Reino et al., 2017) and Tanzania reporting exports of over 500,000 birds of CITES species from 1983 to 1990 (Leader‐Williams & Tibanyenda, 1996). In 2005, the EU, which had been the leading importer of birds from Africa, ended legal imports of wild birds, which reduced the overall volume of global trade in these species (Reino et al., 2017). In 2007, 116 African bird species, which made up much of the trade prior to 2005, were removed from CITES Appendix III (Notification to the Parties No. 2007/007), and, as a result, these species have not been consistently monitored by CITES Parties since. At the 18th CITES Conference of the Parties, parties initiated a process to examine trade in Passeriformes due to concerns about the volumes of trade and noted that the songbird trade from Africa is currently the most poorly understood (CoP18 Doc. 79). Outside CITES, little is known about trade in non‐CITES bird species from this region, although recent research of trader activity on social media platforms indicates substantial exports of a broad diversity of birds from African countries (Davies, Nuno, et al., 2022).

Southeast Asia has been highlighted in several studies as a key region for wildlife trade (Bush et al., 2014; Harris et al., 2017; Marshall et al., 2020). In this region, the Hong Kong Special Administrative Region of the People's Republic of China (hereafter referred to as Hong Kong) and Singapore serve as major nodes in international wildlife trade networks for a number of historical, geographical, and logistical reasons (e.g., Andersson et al., 2021; Chan et al., 2021; Hatten et al., 2020). Trade links established during the colonial era enabled these cities to function as entry points to Asia (specifically the People's Republic of China, in the case of Hong Kong) and to play a central role in international trade for wildlife and wildlife products (ADMCF, 2018; Andersson et al., 2021; Eaton et al., 2017; Webster, 1975). Hong Kong and Singapore are significant trade points in the international live bird trade (Aloysius et al., 2020; Andersson et al., 2021; Chiok et al., 2022; Inglis et al., 2022; Nash, 1993; Poole & Shepherd, 2017). Both were among the top 10 importing countries (excluding European countries) from 1995 to 2011 (Reino et al., 2017); Singapore was the third largest importer after European countries and the United States in 1988 (Leader‐Williams & Tibanyenda, 1996). Both Singapore and Hong Kong have active bird‐keeping traditions (Chan, 2006; Eaton et al., 2017; Nash, 1993). Purchasing birds for mercy release during religious ceremonies is also common (Chan, 2006; KFBG, 2011). Although Asian songbirds, such as munias (*Lonchura* spp.) and white‐eyes (*Zosterops* spp.), have traditionally been the most common species used in these religious release ceremonies (Chan, 2006; KFBG, 2011), African species are also occasionally released (J. A. Allcock, pers. comm. 2024).

We used the UN Comtrade database to describe the volume, dynamics, and direction of trade in non‐CITES bird species from Africa to key trade centers in Southeast Asia from 2006 to 2020. We supplemented these data with more granular species‐specific import data on species from African countries to Hong Kong acquired from the Hong Kong Agriculture, Fisheries and Conservation Department (AFCD) to identify which species are predominant in trade and consider the implications for initiatives to mitigate trade‐related harms. We further compared data availability between exporting and importing countries to explore the challenges and opportunities of using UN Comtrade data for understanding trade in non‐CITES species.

## METHODS

### Comtrade data collection and filtering

Annually, UN member parties report commodity trade to the World Customs Organization, using 5300 globally agreed six‐digit harmonized system (HS) codes to classify different commodities in trade. These data are aggregated in the UN Comtrade database, which is publicly available online at UN Comtrade (Comtrade.un.org). A single trade entry in this database reports exports or imports from a country in each year. To examine the volume and source of trade of live birds for pets between African countries and Southeast Asian trade hubs, we downloaded 427 trade entries using the HS code 10639 (live birds excluding birds of prey, Psittaciformes, ostriches, emus, and live poultry) for 2002–2020, specifying the trade flow as import and the reporter as either China, Hong Kong SAR or Singapore. Because discrepancies have been observed between exporter‐ and importer‐reported volumes (e.g., Chen et al., 2022), a problem that also affects CITES data (Robinson & Sinovas, 2018), we included data only from importers in this study. We excluded 38 entries where the partner was “world,” representing the aggregated imports of all trade partners in that year, to avoid replication, leaving 389 entries. We used HS code 10639 as the best approximation of trade in non‐CITES bird species. The HS code 10639 subsumes over 9000 bird species, of which only 60 (0.6%) represent CITES species native to African countries, such as turacos (Musophagidae) and cranes (Gruidae). To establish that these CITES species did not comprise a large proportion of trade under HS code 10639, we downloaded comparative tabulations of all records of live birds imported to Hong Kong and Singapore from 2006 to 2020 from the CITES Trade Database under all purpose and source codes (downloaded 23 March 2023). These data showed that only nine of these 60 species (which were not removed from the CITES appendices in 2007) were imported in the period, totaling only 1194 individual birds.

In each Comtrade entry, the amount of trade can be reported as quantities, trade value, or net weight. In the 380 entries we downloaded, quantity data were listed in 96.1% of entries (*n* = 374), trade value in 100%, and net weight in 43.7% (*n* = 170). For this analysis, we chose to use quantity in our analyses as our primary metric. *Quantity* is defined as the number of individuals, which we interpreted as the number of individual birds, our primary metric. We chose quantity in part because it is a more direct measure of trade volume than trade value, given the diversity of species and associated value in the bird trade, and it has more conservation relevance. Trade value strongly depends on which species are being traded and the market dynamics at that time. Using quantitative data has some limitations. Reporting could be inconsistent due to varying interpretations of *quantity*, such as reporting the number of shipment boxes or permits issued, and it is unclear whether quantity includes animals that have died in transit. These limitations, which are common to other trade databases such as the CITES Trade Database, mean data should be treated as approximations and interpreted with appropriate caution. Fifteen entries (3.9% of all entries) were not included because they had no quantity unit or value.

China and Hong Kong have a long history of bird trade, with high volumes of birds passing between the two for at least 100 years (Fiennes et al., 2021; Liang et al., 2024; Melville, 1982; Nash, 1993; Wang et al., 2021). We excluded 16 entries between Hong Kong and China from subsequent analyses because the very large volume of imports into Hong Kong from China obscured patterns of trade from other countries. A reported 14,796,692 birds were imported from 2002 to 2017, with a reported trade value of US$28,755,518, accounting for 85.7% of the overall quantity of imports and 97.9% of imports into Hong Kong alone. There were no entries with imports from China to Singapore. Furthermore, we chose not to compare trade before and after the EU trade ban in 2005 and instead only to analyze data after the EU trade ban (2006–2020) (*n* = 268). This is because a considerable proportion of the trade quantity records during the period prior to 2005 were indirect estimations made through adjusting the provided Trade Value using the Standard Unit Value of the product category (CEPII, 2010). Quantities estimated using this approach were unusually high compared with directly reported quantity data.

To compare reporting by importing and exporting countries, we identified the African countries that were trade partners with Singapore or Hong Kong based on import data. For these countries, we downloaded Comtrade data from 2006 to 2020, specifying the HS commodity code as 10639 (“live birds,” excluding birds of prey, Psittaciformes, ostriches, and emus), the trade flow as “export,” and the partner as either “China, Hong Kong SAR” or “Singapore.”

### Species‐specific data collection

To gain insight into the species‐level composition of traded songbirds, we examined import data provided by the AFCD of the Government of Hong Kong for the years 2015–2020, specifying imports of African bird species, excluding parrots and birds of prey. These data included the total number of individuals of each species imported in each year and the exporting countries, though the number of individuals was not disaggregated by country. In 2 years, African species were imported from Malaysia. These data were included because there is a strong chance that these shipments are re‐exports from African countries. Malaysia is a major exporter of live birds (Nijman, 2010) and has no large breeding facilities for taxa included in this study to our knowledge. Species quantities were presented as a range between the quantity from only African countries and the quantity from all countries, including Malaysia, where relevant. Singapore collects similar species‐specific data regarding non‐CITES species based on permits issued. We contacted Singapore customs requesting these data but were not successful in obtaining it.

### Conservation implications

To explore the conservation and population status of traded species and the potential risks of trade, we downloaded data from the International Union for Conservation of Nature (IUCN) Red List of Threatened Species and estimated population trend from BirdLife Datazone (http://datazone.birdlife.org/species/search, downloaded 20 April 2024) and used the Songbirds in Trade Database (SiTDB) (Juergens et al., 2021), which summarizes data from market and social media surveys, peer‐reviewed and avicultural literature, and published and unpublished expert notes, to identify species traded in quantities that could plausibly affect the sustainability of the species or particular populations. This plausible category is assigned in the SiTDB when estimated domestic and international trade is high or extreme relative to population size.

## RESULTS

### Global imports of songbirds to Singapore and Hong Kong

From 2006 to 2020, Hong Kong and Singapore reported total imports of 1,085,326 individual birds from all trade partners, excluding imports from China. Overall trade fluctuated over this period but declined to its lowest levels in 2018 (*n* = 30,430) and 2020 (*n* = 33,030). Singapore was the largest importer, importing 831,765 individual birds, accounting for 76.6% of reported birds during 2006–2020 (Appendix S1).

### General patterns of import from Africa

**FIGURE 1:**
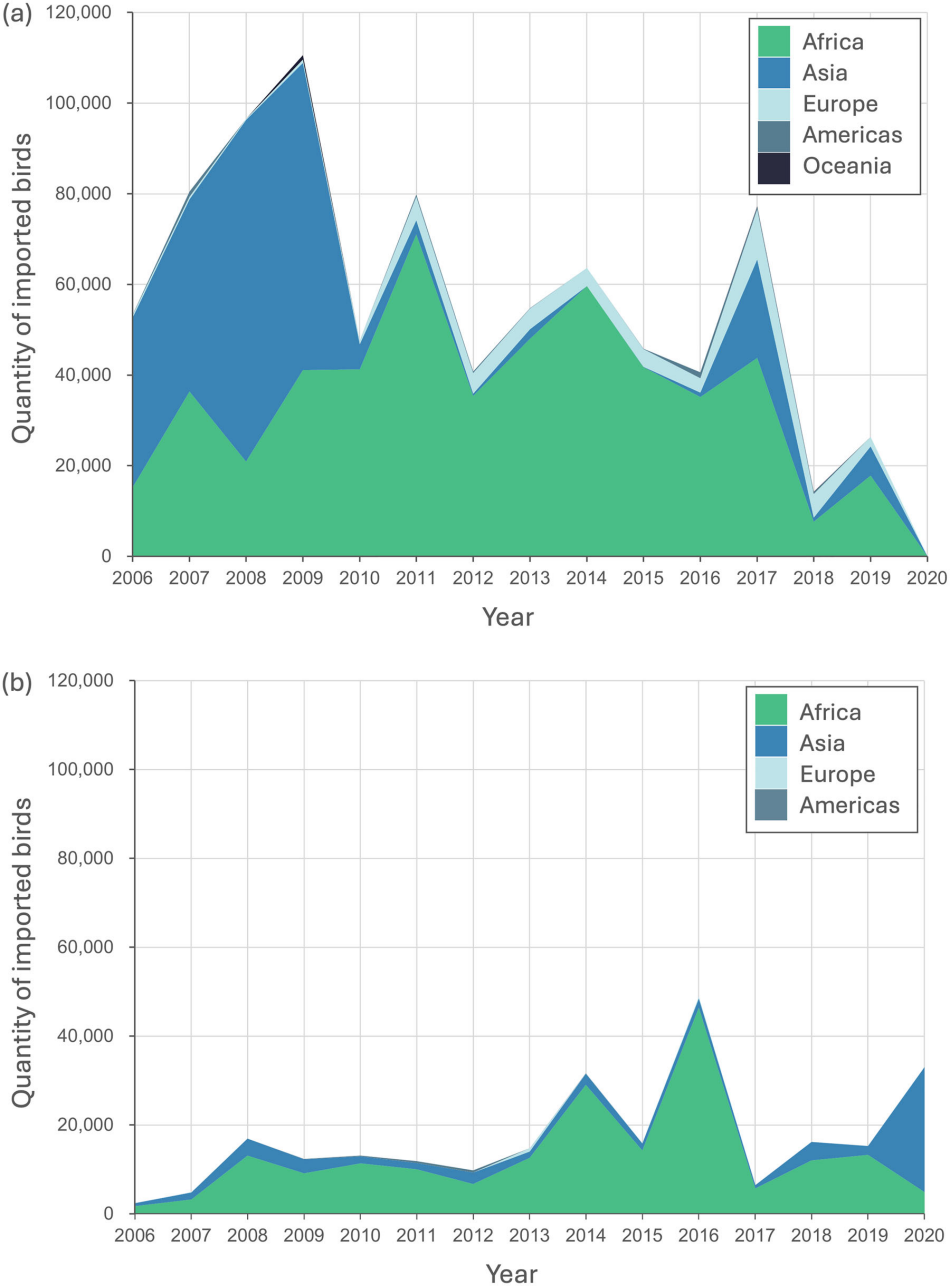
Quantity of live birds (excluding birds of prey, Psittaciformes, ostriches, emus, and live poultry) imported to (a) Singapore and (b) Hong Kong from 2006 to 2020 by world region based on import records for HS code 10639 downloaded from the UN Comtrade database. Imports from China into Hong Kong, which, if included, would account for 97.1% (8,492,083 birds) of imports into Hong Kong in this period, are excluded.

**FIGURE 2:**
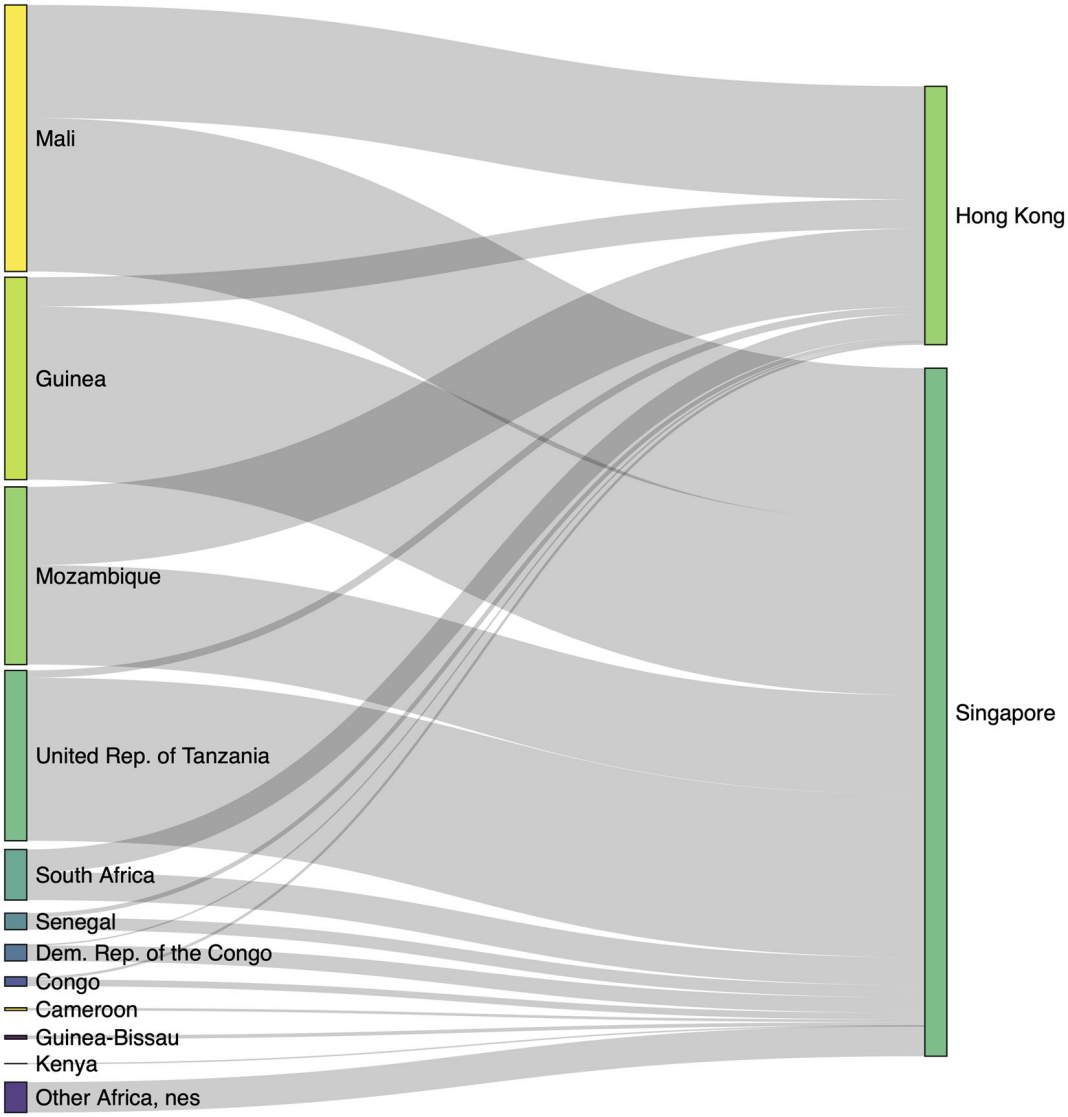
Trade flows of live birds (excluding birds of prey, Psittaciformes, ostriches, emus, and live poultry) from African exporting countries to Singapore and Hong Kong from 2006 to 2020 based on import records for HS code 10639 downloaded from the UN Comtrade database (bar height, relative quantity of birds reported as imported).

**FIGURE 3:**
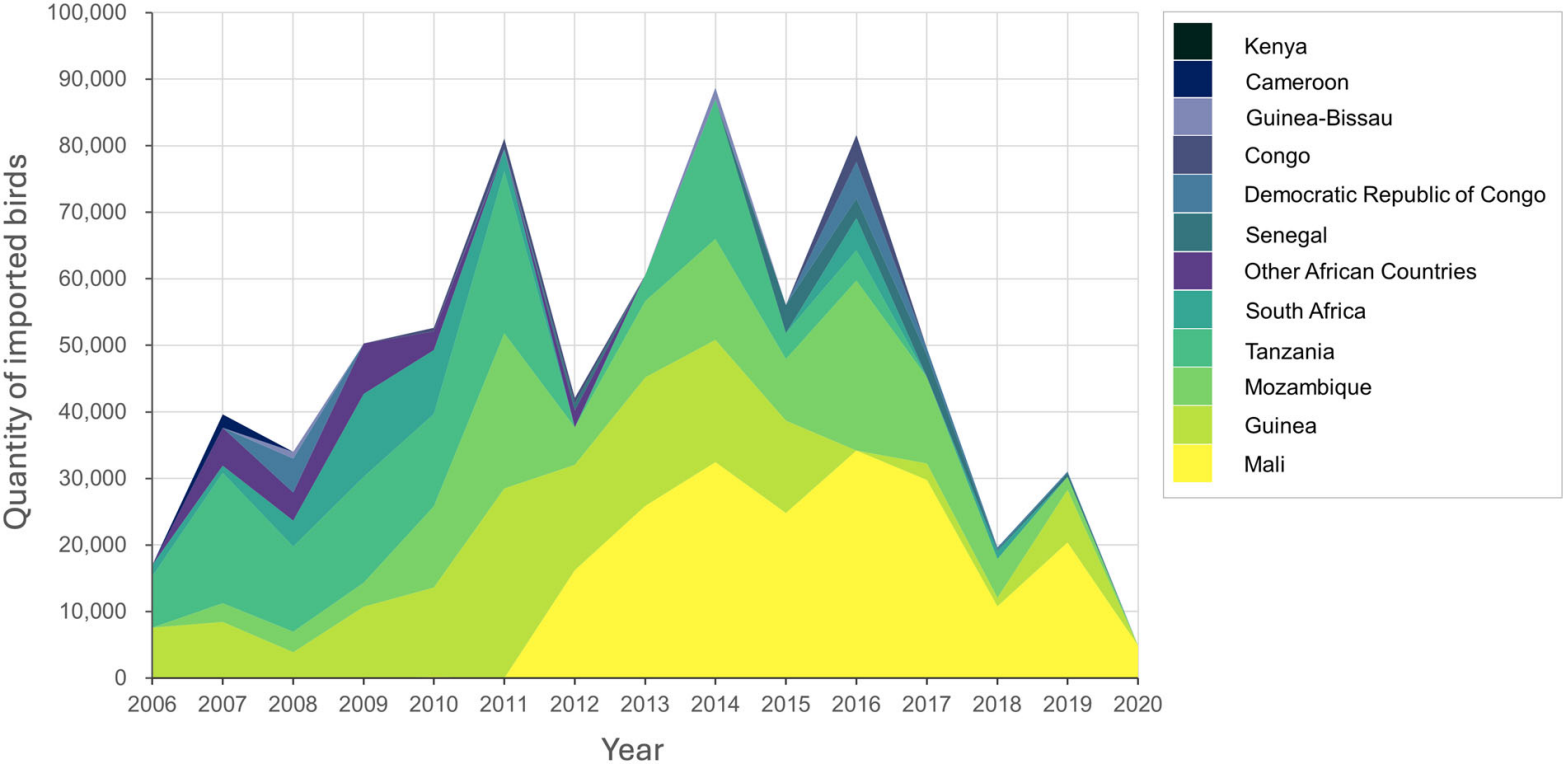
Quantity of live birds (excluding birds of prey, Psittaciformes, ostriches, emus, and live poultry) imported to Hong Kong and Singapore from African countries from 2006 to 2020 based on import records for HS code 10639 downloaded from the UN Comtrade database.

During the period we considered, and excluding trade from China into Hong Kong, Africa was the largest source of birds imported into both Hong Kong and Singapore, accounting for 65.3% of the total reported quantity (*n* = 708,861) (Table 1). The prominence of Africa as a source increased over the period, and from 2010 onward, Africa accounted for 80.2% of imports (Figure 1). Singapore imported 492,507 birds from 11 named African countries, primarily from Mali, Guinea, and the United Republic of Tanzania, which accounted for 71.1% of African bird imports to Singapore, with an additional 22,750 birds imported from unnamed African countries (4.4%) (Figures 2 & 3). Hong Kong imported 193,604 birds from eight named African countries, primarily Mali and Mozambique, which accounted for 74% of African bird imports to Hong Kong (Figure 2). Based on import data, Singapore was a larger trade partner than Hong Kong for all African trade partners.

### Country‐specific patterns

The biggest exporters of wild birds over the entire study period were Mali, Guinea, Mozambique, and the United Republic of Tanzania, together accounting for 86.3% of imports to Hong Kong and Singapore from African countries (*n* = 611,785) (Appendix S2). Mali was the largest trade partner, accounting for 28.2% of all imports from African countries. Mali did not report any exports prior to 2012 and accounted for 45.9% of all imports after 2011. The profile of major exporting countries changed over time. Tanzania and Guinea were the most prominent exporters prior to 2010, and Mali and Mozambique were more prominent after 2011 (Figure 3).

### Comparisons of importer‐reported and exporter‐reported data

There was a difference in the availability of trade entries from importing (*n* = 79) and exporting countries (*n* = 32) (Appendix S1). For the two biggest trading partners, Mali and Guinea, no export data were available. Records with quantity were found for 12 entries, six from Mozambique and six from South Africa, which reported total exports of 31,784 individual birds from 2006 to 2020. This represents a fraction of the 170,772 birds reported imported by Singapore and Hong Kong from Mozambique and South Africa.

### Hong Kong species‐specific import data

For 2015–2020, Hong Kong AFCD data indicated that from 132,905 to 147,908 birds representing 34 African species were imported from five African countries (Mali, Mozambique, Senegal, Tanzania, and Guinea) and Malaysia, all of which were songbirds (Passeriformes) (Appendix S2). There were 26 species imported only from African countries. Two species (common waxbill [*Estrilda astrild*] and Cameroon indigobird [*Vidua camerunensis*]) were imported only from Malaysia, and six species were imported from both African countries and Malaysia. Two species dominated imports from 2015 to 2020: yellow‐fronted canary (*Crithagra mozambica*) (66,583–66,883 individuals) and white‐rumped seedeater (*Crithagra leucopygia*) (45,990–57,590 individuals), which together accounted for 84% of recorded imports from Africa from 2015 to 2020. More broadly, the top six species came from the genus *Crithagra* (canaries) and accounted for 95.1% of all birds imported from Africa in this period. All species were categorized as least concern by the IUCN, but wild populations were considered declining in four species (yellow‐fronted canary, lemon‐breasted canary [*Crithagra citrinipectus*], violet‐backed starling [*Cinnyricinclus leucogaster*], and orange‐breasted sunbird [*Anthobaphes violacea*]) and unknown in one species (emerald starling [*Lamprotornis iris*]). Estimates based on the Songbirds in Trade Database showed that trade is a plausible threat to wild populations of six of these species imported to Hong Kong (Appendix S3).

## DISCUSSION

Our analyses of legal trade in birds between Africa and two Asian trade hubs reported in the UN Comtrade database indicated that Africa has been a significant source of non‐CITES live birds to these two key global trade hubs in Asia since 2010. Imports primarily came from a small number of countries in West and East Africa, but the countries involved shifted during the study period and also differed from the period prior to 2006, indicating dynamic trade patterns potentially responding to changes in availability, demand, transport infrastructure, and regulation.

### Trade volume and dynamics

A comparison of Comtrade data with concurrent CITES data suggested that analyses of international live bird trade based on CITES data alone may severely underestimate the true volume of trade (e.g., Harfoot et al., 2018; Reino et al., 2017). The Comtrade data we analyzed indicated that Singapore imported 831,765 birds (excluding parrots, birds of prey, ostriches, and emus) over 15 years (2006–2020), which is more than three times greater than estimated imports of CITES species to Singapore from countries globally in 2005–2014, based on CITES importer‐reported data (*n* = 225,561) (Poole & Shepherd, 2017). Even this is likely an underestimate of the true volume of trade due to limitations in the reporting of Comtrade data (see “Opportunities and challenges of UN Comtrade data”).

The high volume of imported birds reflects a broader trend, identified in other literature, of Asia acting as a major center of demand in the exotic live bird trade (Bush et al., 2014; Chan et al., 2021). For example, imports of CITES‐listed bird species into Bangladesh and Pakistan have increased dramatically in the last decade (Poonia et al., 2022), and permits issued for non‐CITES birds for import into Bangladesh suggest high import volumes of a diverse array of exotic species, with permits issued for 167,186 birds representing 269 species from October 2020 to September 2021 (Davies & Martin, 2022). Growth in demand for live birds in Asia could have a significant impact on wild bird populations and could increase the risk of disease transmission and poor welfare without corresponding growth in management and enforcement capacity (Eaton et al., 2015; Leupen et al., 2020; Sykes, 2017). The role of Africa as a major source of birds for trade in Southeast Asian countries has not been well quantified, but our data suggested that Africa has been an important exporter for several decades at least (see also Melville [1982] and Nash [1993]). A complete understanding of shifting patterns of trade requires the consideration of data on bird imports beyond CITES species from all importing countries, making use of multiple sources, including customs and environmental government departments.

Comtrade data indicated a drop in imports in 2020 that may reflect the impact of the COVID‐19 pandemic and increased restrictions on international travel (Wildlife Justice Commission, 2020). However, the data also suggested that imports had been low since 2018. Possible explanations for this decline in imports include export restrictions (e.g., Tanzania implemented a national ban on wildlife exports in 2016 [Tanzania Government Notice 181 of, 2016] that coincided with the last year Tanzanian exports were reported in the data); import restrictions (e.g., Singapore prohibits imports of ornamental birds from countries with recent avian influenza outbreaks [Animal & Veterinary Services, 2021]); and declines in wild populations in areas of heavy trapping, although data on the status of wild populations of most exploited species are not known due to a lack of monitoring (Martin, 2018a).

### Importers and exporters

Prior to 2006, Mali and Guinea were leading exporters of African birds and, along with Senegal, exported more birds listed as CITES species than any other country (Reino et al., 2017). Our analyses of the Comtrade data indicated that these countries continue to be prominent exporters of birds. As the largest source of traded birds from Africa, the role of Mali in the bird trade particularly warrants investigation. Mali has been implicated in the possible misuse of export permits to export threatened parrots not found in the country (CITES, 2019; Martin, 2018b) and has acted as a transit destination for exports of CITES‐listed parrots from South Africa (CITES Trade Database [CITES, 2022]). It is unclear whether similar trade relationships and permitting issues also exist in non‐CITES species. Senegal was identified as an exporter of white‐rumped seedeater and yellow‐fronted canary in AFCD data but was surprisingly a comparatively smaller export partner to both Hong Kong and Singapore despite being one of the largest historical exporters of birds from the region (Leader‐Williams & Tibanyenda, 1996; Ruelle & Bruggers, 1983). It is possible that exports from Senegal have been overlooked by our study if Senegal has established trade partnerships with countries other than Singapore and Hong Kong. Recent surveys of birds offered for sale on social media suggest a diverse range of wild‐sourced bird species are still exported from Senegal (Davies, Nuno, et al., 2022). There are no recent data of which we are aware on the current extent of bird trapping for international trade in Mali, Guinea, or Senegal, making it difficult to determine whether the industries remain diminished, following the removal of their primary markets in the EU, or whether they have recovered following the establishment of connections to new trading partners. East African countries, Tanzania and Mozambique, were both prominent trade partners. This was surprising as recent literature has emphasized the role of West African countries in the African live bird trade (Davies, D'Cruze, et al., 2022; Davies, Nuno, et al., 2022; Reino et al., 2017). Tanzania was one of the leading exporters of birds globally in the 1980s (Leader‐Williams & Tibanyenda, 1996), but little has been published on the recent role of East African countries in global bird trade, and the scale and scope of this trade, particularly regarding Mozambique, demand further investigation.

### Species‐level analyses

Bird imports into Hong Kong over the period examined, based on AFCD data, were dominated by songbirds (Passeriformes), particularly the yellow‐fronted canary and the white‐rumped seedeater. This is largely consistent with historical data on the African bird trade (Leader‐Williams & Tibanyenda, 1996; Nash, 1993; Ruelle & Bruggers, 1983), historical CITES data (Reino et al., 2017; Supporting Information), and online monitoring (Davies, Nuno, et al., 2022). Although many imported species are listed as least concern on the IUCN Red List of Threatened species, there are little recent available field data on abundance, distribution, or population trends in source countries with which to determine the sustainability of trade (Martin, 2018a).

Among the most commonly imported species, two species (yellow‐fronted canary and lemon‐breasted canary) are considered in decline as a direct result of trapping for trade, and for two species (yellow‐fronted canary and black‐throated canary [*Crithagra atrogularis*]), trade is identified as a plausible threat in the SiTDB (Juergens et al., 2021), with the most popular species, the yellow‐fronted canary, included in both categories. Although there are few data with which to determine whether these species are wild sourced or captive bred (and Comtrade codes do not specify this information), we consider it likely that canaries exported from these countries are wild sourced, given the likely price differentials between production in captivity and capture from the wild and regulations permitting the capture and export of these species (Leader‐Williams & Tibyanyeda, 1996). The majority of reported exports of CITES‐listed bird species native to the countries included in our study are wild sourced, despite much of this trade involving species that can be, and are, readily bred in captivity in other countries. It seems unlikely that a contrary pattern would exist among non‐CITES birds. Furthermore, a recent review of wildlife farming globally did not identify any bird breeding facilities in any of the source countries other than South Africa (Green et al., 2023). There are numerous examples of once common and widespread songbird species experiencing severe population declines and range retraction as a result of overexploitation, suggesting the conservation community should not be complacent about impacts on wild populations (e.g., Khelifa et al., 2017; Leupen et al., 2020). Current data on the population status of popular African songbirds, as well as other bird groups, particularly from major exporting countries, are urgently needed to assess the sustainability and impacts of this ongoing trade.

### Potential implications of trade for importing countries

Both Hong Kong and Singapore act as major wildlife trade hubs. The low volumes of non‐CITES‐listed African birds in Singapore markets (Chiok & Chng, 2021; Eaton et al., 2017) and for sale in Hong Kong markets (Chan, 2006, Dingle et al., 2026), in contrast to Taiwan bird markets (Su et al., 2014), suggest that either there is very large‐scale misdeclaration of species identity or the majority of birds imported are not retained in domestic markets and are re‐exported. According to CITES data, Singapore re‐exports wildlife products to many countries, including Taiwan, Japan, and the Netherlands (Shepherd et al., 2012; Su et al., 2014). Observations by local researchers and the intended destination of seized illegal consignments indicate that wildlife is illegally re‐exported from Hong Kong into China (ADMCF, 2018; Inglis et al., 2022).

Bird trade has been linked to the spillover of several diseases, including H5N1, psittacosis, and avian malaria, which affect both humans and naïve wildlife in the regions where trade occurs (Can et al., 2019; Das et al., 2020; Kozuki et al., 2020; Travis et al., 2011). As major trade transit points for large numbers of wild birds, Hong Kong and Singapore might be at particularly high risk from the spillover of infectious diseases. Multiple strains of avian influenza have been identified in sub‐Saharan countries, including in species trapped for the international live bird trade (Kalonda et al., 2020). Singapore has enacted policies to restrict imports of birds from countries experiencing outbreaks of avian influenza with the aim of preventing the spread of H5N1 (Animal & Veterinary Services, 2021). It could be prudent for importing countries to mitigate biosecurity risks by broadening restrictions on imports of live birds to include other exporting countries unable to demonstrate adequate surveillance for pathogens of human and animal health concern and by implementing robust quarantine procedures.

### Opportunities and challenges of UN Comtrade data

Customs and import data offer an alternative source of data that can contextualize, complement, and highlight limitations in other research methods, such as social media and market surveys. The strong live bird trade connection between African countries and Hong Kong and Singapore was not apparent from previous studies based on CITES data and social media studies (Martin et al., 2018; Reino et al., 2017). Our study serves as a note of caution that overreliance on any single data source may paint a misleading picture of trade patterns. For instance, social media studies may only capture certain country connections, depending on the extent to which platforms are used in those countries or whether trade in particular taxa is facilitated online.

**TABLE 1:** Total number of import records and quantity of live birds (excluding birds of prey, Psittaciformes, ostriches, emus, and live poultry) (HS code 10639) imported to Hong Kong (excluding imports from China) and Singapore by world region from 2006 to 2020, recorded in the UN Comtrade database.

Reporting country	Number of entries	Quantity					
		Africa	Asia	Europe	Americas	Oceania	total
Hong Kong	80	193,604	57,380	1358	1219	–	253,561
Singapore	188	515,257	264,303	46,543	4406	1256	831,765
Total	268	708,861	321,683	47,901	5625	1256	1,085,326

Although Comtrade data can provide insights into the scale and scope of trade among groups of species, it has limited application for understanding trade at lower taxonomic (i.e., species) levels. We demonstrated how these limitations can be partially mitigated with the support of supplementary data from other sources, such as the data from the Hong Kong AFCD. However, such data are not readily accessible, and we were unable to source equivalent data from Singapore. This challenge can be overcome, and the value of Comtrade data for understanding wildlife trade improved, through increasing the taxonomic specificity of standard Comtrade data by distilling existing HS codes and establishing genus‐specific codes, particularly for broad categories including rare species, such as “other birds” (Andersson et al., 2021; Chan et al., 2015), as has been done with HS code 030191 (“other live fish” category), in which species of high concern have been assigned their own unique HS code. However, as with other trade data sources, misdeclaration either by accident or intentionally in order to traffic other species (Martin et al., 2019) may mean that some species are disproportionately represented or omitted.

An important limitation of the Comtrade data is a lack of reporting consistency between importing and exporting countries. A comparison of available Comtrade data from Hong Kong and Singapore with exporting African countries revealed a wide disparity in the number of entries and reported volume for all partners. This means that the volume of trade cannot be verified with the exporter, and there are no available data on the relative size of Hong Kong and Singapore as bird importers for African countries. This information is particularly important for understanding the broader picture of trade in non‐CITES‐listed species and for evaluating the impact of recent versus historical trade. The complete absence of data from the two largest exporters, Mali and Guinea, is particularly concerning. Similar discrepancies have been observed in other areas of wildlife trade, including sea cucumbers and fish maws (Constant et al., 2020; Louw & Bűrgener, 2020). The reasons for omission are unclear, although possible explanations include ineffective reporting, low customs capacity, or incorrect use of HS codes, either accidentally or intentionally, to avoid taxes and tariffs.

### Recommendations

Our study revealed substantial trade, involving over a million birds, between Africa and two key Asian trade hubs, Hong Kong and Singapore, over the last 15 years. The vast majority of these birds were likely captured from the wild, with potential implications for the sustainability of trade, the overexploitation of wild populations, and biosecurity risks to people and animals. Although multiple countries have enacted measures to prohibit or severely curtail both imports and exports of wild‐sourced birds (Gilardi, 2006), record keeping and monitoring of trade in species not listed on any of the CITES appendices remain minimal. No coordinated global monitoring of the scale and scope of this trade currently takes place, and there is a dearth of information on the numbers of individuals from different species taken from the wild and the consequences for wild populations, ecosystems, and human health. Comtrade data offer one route for collecting more data on volumes of bird trade. Although currently Comtrade data do not provide sufficient resolution to track individual species, increased resourcing to build capacity for finer‐scale reporting of Comtrade data would increase our ability to monitor and investigate international wildlife trade (Constant et al., 2020; Louw & Bűrgener, 2020). Ongoing monitoring of trade should therefore include data from multiple sources. The HS codes could be revised to include more specific codes to genus or species level, as has been done for several species of highly traded fish species (FAO, 2022), which would improve the ability to track trade volumes (Chan et al., 2015). A recent study of global legal trade specifically highlighted that more specific HS codes would be particularly valuable for tracking bird trade (Andersson et al., 2021). Although improving data collection within any one database is necessary, our results highlight the importance of including multiple datasets when evaluating trade levels. Each database comes with its own limitations and biases, such that relying on a single source of data may lead to inaccurate evaluations of the extent of bird trade. Comtrade data provide information on trade volumes of all bird species and therefore can be used to supplement existing CITES data, which only include CITES‐listed (and therefore threatened) species, and market data, which generally only provide a snapshot of trade during survey dates.

## Supporting information



Appendix S1. The total number of import records and quantity of birds imported to Hong Kong and Singapore by world region in the period 2006‐2020 (from HS Codes).
Appendix S2. The number of trade records and quantity of birds reported as imported by Hong Kong and Singapore and reported as exported by exporting countries for the five largest African exporters based on import records.
Appendix S3. Species, conservation status, estimation of whether trade is threat to populations in the Songbirds in Trade Database (SiTDB) and quantity of birds imported into Hong Kong in the period 2015‐2020, from both African and non‐African countries, according to import data sourced from the Agriculture, Fisheries and Conservation Department (AFCD).

Supplementary Information
