# Supplementary material for: Using customs data to understand overlooked trade in non‐CITES birds between Africa and Asia
Source: Conserv Biol. 2026 Mar 23;40(4):e70265. doi: 10.1111/cobi.70265 (PMC13392723; doi:10.1111/cobi.70265)
Supplement: Supplementary file 1 — Appendix S1. The total number of import records and quantity of birds imported to Hong Kong and Singapore by world region in the period 2006‐2020 (from HS Codes). Appendix S2. The number of trade records and quantity of birds reported as imported by Hong Kong and Singapore and reported as exported by exporting countries for the five largest African exporters based on import records. Appendix S3. Species, conservation status, estimation of whether trade is threat to populations in the Songbirds in Trade Database (SiTDB) and quantity of birds imported into Hong Kong in the period 2015‐2020, from both African and non‐African countries, according to import data sourced from the Agriculture, Fisheries and Conservation Department (AFCD). [file COBI-40-e70265-s002.docx]

**Appendix S1.** The total number of import records and quantity of birds imported to Hong Kong and Singapore by world region in the period 2006-2020 (from HS Codes).

| **Reporting Country** | **No. Entries** | **Quantity** | | | | | |
| --- | --- | --- | --- | --- | --- | --- | --- |
|  |  | **Africa** | **Asia** | **Europe** | **Americas** | **Oceania** | **Total** |
| Hong Kong | 80 | 193,604 | 57,380 | 1,358 | 1,219 | - | 253,561 |
| Singapore | 188 | 515,257 | 264,303 | 46,543 | 4,406 | 1,256 | 831,765 |
| **Total** | **268** | **708,861** | **321,683** | **47,901** | **5,625** | **1,256** | **1,085,326** |

**Appendix S2.** The number of trade records and quantity of birds reported as imported by Hong Kong and Singapore and reported as exported by exporting countries for the five largest African exporters based on import records.

| **Country** | **Reported Imports** | | | | **Reported Exports** | |
| --- | --- | --- | --- | --- | --- | --- |
|  | **No. Entries** | **Quantity** | | | **No. Entries*** | **Quantity** |
|  |  | **Hong Kong** | **Singapore** | **Total** |  |  |
| Mali | 18 | 84,749 | 114,927 | 199,676 | 1(0) | - |
| Guinea | 21 | 21,890 | 129,771 | 151,661 | - | - |
| Mozambique | 20 | 58,595 | 74,251 | 132,846 | 9 (6) | 29,260 |
| United Rep. of Tanzania | 13 | 5,513 | 122,089 | 127,602 | 9 (0) | - |
| South Africa | 15 | 16,970 | 20,956 | 37,926 | 10(6) | 2,524 |
| Senegal | 7 | 3,250 | 9,150 | 12,400 | 3 (0) | - |
| Democratic Rep. of Congo | 7 | 700 | 11,618 | 12,318 | - | - |
| Congo | 5 | 1,937 | 5,000 | 6,937 | - | - |
| Guinea-Bissau | 2 | - | 2,735 | 2,735 | - | - |
| Cameroon | 1 | - | 2,000 | 2,000 | - | - |
| Kenya | 1 | - | 10 | 10 | - | - |
| Total | 79 | 193,604 | 515,257 | 708,861 | 32(12) | 31,784 |

* The number in brackets indicated the number of entries that provided quantity in number of items

**Appendix S3.** Species, conservation status, estimation of whether trade is threat to populations in the Songbirds in Trade Database (SiTDB) and quantity of birds imported into Hong Kong in the period 2015-2020, from both African and non-African countries, according to import data sourced from the Agriculture, Fisheries and Conservation Department (AFCD). All species are categorised as Least Concern by the International Union for Conservation of Nature (IUCN). Red text indicates individuals imported from Malaysia.

| **Common Name** | **Scientific Name** | **Population Status** | **SiTDB**  **Trade as Threat** | **Exporting Countries** | **Quantity Imported** | | | | | | |
| --- | --- | --- | --- | --- | --- | --- | --- | --- | --- | --- | --- |
|  |  |  |  |  | **2015** | **2016** | **2017** | **2018** | **2019** | **2020** | **Total** |
| Yellow-fronted Canary | *Crithagra mozambica* | Declining | Plausible | Mali, Mozambique, Senegal, Tanzania, Guinea, Malaysia | 19,770 | 13,230 | 9,330 | 11,900 | 9,250 | 3,403* | 66,883 |
| White-rumped Seedeater | *Crithagra leucopygia* | Stable | - | Mali, Senegal, Guinea, Malaysia | 11,600** | 12,460 | 7,580 | 12,400 | 12,550 | 1,000 | 57,590 |
| Yellow-rumped seedeater | *Crithagra atrogularis* | Stable | Plausible | Mozambique, Tanzania, Mali, Malaysia | 2,660 | 350 | 500 | 1,700 | 800 | 50 | 6,060 |
| Yellow canary | *Crithagra flaviventris* | Stable | - | Mozambique, Malaysia | 600 | 1,650 | 650 | 1,250 | - | 100 | 4,250 |
| Lemon-breasted seedeater | *Crithagra citrinipectus* | Declining | - | Mozambique, Mali, Malaysia | 1,275 | 960 | 1,000 | - | 200 | 50 | 3,485 |
| Brimstone canary | *Crithagra sulphurata* | Stable | - | Mozambique, Tanzania, Mali | 600 | 560 | 400 | 600 | 200 | - | 2,360 |
| Long-tailed paradise whydah | *Vidua paradisaea* | Stable | - | Mali, Mozambique | 400 | 650 | - | 400 | 280 | 150 | 1,880 |
| Red-cheeked Cordon-bleu | *Uraeginthus bengalus* | Stable | - | Guinea, Mali, Malaysia | 300 | - | 220 | 80 | 400 | - | 1,000 |
| Southern red bishop | *Euplectes orix* | Stable | - | Guinea, Mali | - | - | - | 300 |  | 260 | 560 |
| Yellow bishop | *Euplectes capensis* | Stable | - | Guinea, Mali | - | - | - | 300 | - | 150 | 450 |
| Lavender waxbill | *Estrilda caerulescens* | Stable | - | Mali | - | - | - | - | 300 | - | 300 |
| Zebra waxbill | *Amandava subflava* | Stable | - | Guinea, Mali | - | - | 290 | - | - | - | 290 |
| Village indigobird | *Vidua chalybeata* | Stable | - | Mozambique, Mali | - | 150 | - | - | - | 130 | 280 |
| Red-billed firefinch | *Lagonosticta senegala* | Stable | - | Mali | - | - | - | - | 250 | - | 250 |
| Sudan golden sparrow | *Passer luteus* | Stable | - | Mali | 200 | - | - | - | - | 50 | 250 |
| Black-headed canary | *Serinus alario* | Stable | - | Mozambique | - | 150 | 100 | - | - | - | 250 |
| Cut-throat finch | *Amadina fasciata* | Stable | - | Mali | - | - | - | - | 150 | 70 | 220 |
| Cameroon Indigobird | *Vidua camerunensis* |  |  | Malaysia | 200 | - | - | - | - | - | 200 |
| Pin-tailed whydah | *Vidua macroura* | Stable | Plausible | Mali | 200 | - | - | - | - | - | 200 |
| Bronze mannikin | *Spermestes cucullata* | Stable | - | Mali | - | - | - | - | 200 | - | 200 |
| Orange-cheeked waxbill | *Estrilda melpoda* | Stable | - | Mali | - | - | - | - | 150 | - | 150 |
| Black-rumped waxbill | *Estrilda troglodytes* | Stable | - | Mali | - | - | - | - | 150 | - | 150 |
| Northern yellow white-eye | *Zosterops senegalensis* | Stable | Plausible | Mali | - | - | - | 50 | - | 60 | 110 |
| Common Waxbill | *Estrilda astrild* |  |  | Malaysia | 100 | - | - | - | - | - | 100 |
| Violet-backed starling | *Cinnyricinclus leucogaster* | Declining | Plausible | Guinea, Mali | - | - | 50 | - | - | 20 | 70 |
| Green twinspot | *Mandingoa nitidula* | Stable | - | Mali | - | - | 70 | - | - | - | 70 |
| Emerald starling | *Lamprotornis iris* | Unknown | Plausible | Guinea, Mali | - | - | 20 | 20 | - | 20 | 60 |
| Red-headed finch | *Amadina erythrocephala* | Stable | - | Mali | - | - | - | - | - | 50 | 50 |
| Long-tailed glossy starling | *Lamprotornis caudatus* | Stable | - | Mali | - | - | - | 50 | - | - | 50 |
| Purple glossy starling | *Lamprotornis purpureus* | Stable | - | Guinea | - | - | 50 | - | - | - | 50 |
| Village weaver | *Ploceus cucullatus* | Stable | - | Mali | - | - | - | - | - | 40 | 40 |
| Snowy-crowned robin-chat | *Cossypha niveicapilla* | Stable | - | Guinea | - | - | - | 20 | - | - | 20 |
| Yellow-crowned gonolek | *Laniarius barbarus* | Stable | - | Guinea | - | - | - | 20 | - | - | 20 |
| Orange-breasted sunbird | *Anthobaphes violacea* | Declining | - | Guinea | **-** | **-** | **-** | 10 | **-** | **-** | 10 |
| Total |  |  |  |  | **37,905** | **30,160** | **20,260** | **29,100** | **24,880** | **5,603** | **147,908** |

*Includes 300 individuals imported from Malaysia.

**In 2015, the total number of imports includes birds imported from Malaysia as well as from Mali and Senegal. The data are such that we cannot determine how many of the birds were imported from each country.
